# What do peer support workers do? A job description

**DOI:** 10.1186/1472-6963-12-205

**Published:** 2012-07-19

**Authors:** Nora Jacobson, Lucy Trojanowski, Carolyn S Dewa

**Affiliations:** 1 6767 Frank Lloyd Wright Avenue, Middleton, WI, 53562, USA; 2 Centre for Research on Employment and Workplace Health, Centre for Addiction and Mental Health, 455 Spadina Avenue, Suite 300, Toronto M5S 2G8, Canada; 3 Department of Psychiatry, University of Toronto, 250 College Street, Toronto M5T 1R8, Canada

**Keywords:** Peer support, Job description, Participatory evaluation, Mixed methods

## Abstract

**Background:**

The extant literature suggests that poorly defined job roles make it difficult for peer support workers to be successful, and hinder their integration into multi-disciplinary workplace teams. This article uses data gathered as part of a participatory evaluation of a peer support program at a psychiatric tertiary care facility to specify the work that peers do.

**Methods:**

Data were gathered through interviews, focus groups, and activity logs and were analyzed using a modified grounded theory approach.

**Results:**

Peers engage in direct work with clients and in indirect work that supports their work with clients. The main types of direct work are advocacy, connecting to resources, experiential sharing, building community, relationship building, group facilitation, skill building/mentoring/goal setting, and socialization/self-esteem building. The main types of indirect work are group planning and development, administration, team communication, supervision/training, receiving support, education/awareness building, and information gathering and verification. In addition, peers also do work aimed at building relationships with staff and work aimed at legitimizing the peer role. Experience, approach, presence, role modeling, collaboration, challenge, and compromise can be seen as the tangible enactments of peers’ philosophy of work.

**Conclusions:**

Candidates for positions as peer support workers require more than experience with mental health and/or addiction problems. The job description provided in this article may not be appropriate for all settings, but it will contribute to a better understanding of the peer support worker position, the skills required, and the types of expectations that could define successful fulfillment of the role.

## Introduction

With the advent of the recovery movement [1], interest in peer support has grown in many mental health systems. Particular attention is being paid to collaborative models like peer-run services operated in tandem with traditional services and the employment of peer support workers in clinical environments dominated by the professional disciplines [2-7]. Together, a number of studies suggest that peer support helps people become more engaged and empowered; that it can reduce symptoms and hospitalization; that it is feasible and beneficial to combine it with services provided by disciplinary professionals; and that it improves the subjective well-being of both the people who receive it and the people who provide it [5,6,8-24].

An ongoing area of investigation has been to understand what makes peer support work. Among the components of peer support that have been identified as important are the personal characteristics of peers [25], the values embodied by peer support [9,26,27], the specific activities that fall under the peer support umbrella [2,28], and the processes through which peer support works to effect good results [4,7,9,26,29-31]. Despite the existence of quite a large literature that explores such components, several authors have called for more detailed empirical examination of these “critical ingredients” of peer support [19,22,32]. Such specification would help to address two issues the literature identifies as problematic for peer support: a lack of clarity in peer role expectations and a need for peers to be better integrated into their workplace teams [4,33-35].

Using data gathered as part of an evaluation of a hospital-based peer support program, this article examines the critical ingredients of peer support work by exploring the types of work peers do and how peers do this work. It then proposes a job description for the peer support role. By clearly articulating a detailed set of expectations and qualifications, this description should aid organizations that are developing their own peer support programming.

## Background

A job description is a written description of what the person holding a particular job is expected to do, how they must do it, and the rationale for required job procedures [36]. Accurate descriptions are essential to the success of the incumbent in his/her job because they help to ensure that the recruitment and selection process is executed effectively and that the most qualified candidate is selected for the job. They also serve to guide the goals and activities of the incumbent once he/she is hired. Job descriptions are developed by means of a job analysis, or the process of collecting and analyzing information about a job, including data on job duties, responsibilities, and context, as well as critical ingredients like required competencies and characteristics.

The extant literature suggests that poorly defined job descriptions make it difficult for peers to be successful, and hinder their integration into multi-disciplinary workplace teams. In their study of 27 social service agencies providing mental health services in New York City, 18 of which currently employed peers, Gates and Akabas [33] sought to determine what kinds of procedures, policies and structures would support the contribution of peers to the mental health service system. They found that role conflict and confusion made it difficult for peer and non-peer staff to work together. This role conflict and confusion were the result of poorly operationalized or defined job tasks. When written job descriptions provided by the human resources department were contrasted with the list of tasks actually performed by the peers, it was clear that expectations for peers were much greater than the formal job responsibilities, and were often unreasonable. Gates and Akabas [33] recommend the establishment of structures, policies and practices to guide peer recruitment and to better define the peer position.

In their study of two peer consumer advocate positions on a professional multidisciplinary team, Dixon et al. [4] found that the vagueness and insufficient structure of the consumer advocate role meant that peers were not sure about what they should be doing. Manning and Suire [35] found that having a clearly defined job description provided concrete expectations for peers, and having clear role expectations was critical to employment success. In situations where positions lacked a job description, many peers felt unprepared and were left on their own to figure out what they should do. Moll et al. [34] examined six programs that integrated peer support into traditional mental health services. Among the challenges experienced by peers was the definition and establishment of roles, and a lack of clarity of the work role, particularly at the beginning. The peer positions in each program had a job description, but the role evolved over time and developed as the programs became more familiar with what the incumbent peer was able to do. Gates and Akabas [33] found that role conflict and confusion seemed to occur when non-peer staff were not sufficiently prepared by the agencies to receive a peer colleague to their staff. Dixon et al. [4] also found that the vagueness of the peer role meant that staff members sometimes were not clear of how they should relate to the peer.

The peer support program described in this article is based at a large psychiatric tertiary care hospital. The program places individuals who have experienced mental health and/or addiction challenges as staff members in the facility’s inpatient and outpatient clinical programs, including several programs that are located in the community. As portrayed in its statement of vision and values, the purpose of the peer program is “to enhance recovery, client centered practice, diversity, advocacy and a holistic view of health through lived experience and shared intentional learning.” The program is grounded in Shery Mead’s intentional peer support model, described as being focused on “relational change; a commitment to mutuality, negotiation, noticing power dynamics, and a transparent agreement that both people are there to learn through the process of their relationship” [37].

According to a fact sheet produced by the program’s developers, peers are expected to “[work] with clients as a coach, connector, and partner to support and bridge people back into the community.” The peer role is explicitly a non-clinical one, which “does not involve treatment, assessment or evaluation.” Peers are expected to act as educators and advocates, promoting the program values of autonomy, diversity, and empowerment, and as knowledgeable brokers who are able to link clients to community-based supports and other resources. Peers receive training upon hiring, and some have had opportunities for further education. Peers are supervised both by their unit managers and by an advanced practice clinician who serves as a mentor to the group of peers. In addition, on each unit, program developers designated a “recovery facilitator,” a disciplinary staff member with special interest and training in recovery, to serve as an “intentional ally” to the peer.

The hospital’s peer program was initiated in fall of 2007, with the hiring of one peer. During 2008, eight more peers were hired. Late that year, the program developers approached the authors of this paper with a request to develop a formative evaluation of the peer program in order to generate evidence that could be used to guide the program’s evolution.

## Methods

In keeping with the philosophy of the peer program, and consistent with recommendations in the literature [24,38], the evaluation used a participatory approach that drew upon the expertise of those individuals who had grounded experience of the program as it was actually operating. The main means of participation was through an advisory committee composed of peers, program developers, and researchers. The committee met regularly during the course of the evaluation, working together to determine a research focus, develop data collection instruments, assist in the recruitment of study participants, provide feedback on preliminary findings, and craft recommendations. All data collection and initial analysis activities were carried out by the authors of this paper, who had overall responsibility for the conduct of this evaluation, and a research interviewer.

At early meetings of the advisory committee, a decision was made to focus on two research questions: What are the peers doing? What impact are the peers having? In the course of conducting the evaluation a third question emerged: What challenges are the peers facing? Although this question was not articulated by the advisory committee when the project was planned, it turned out that understanding these challenges was crucial to understanding both the work being done by peers and its impact.

The evaluation used a mixed methods approach. To explore the question of what peers were doing, peers were invited to complete an activity tracking log on two randomly selected days during the study period. The log collected data on time spent by the peers on different types of work. It also allowed them to enter notes providing details about their activities. The logs were completed by 6 peers. (In order to ensure anonymity among a small group, no demographic data were collected from the peers.) A total of 11 logs were completed.

More information about peers’ work, including perceptions of the impact they were having, was gathered using interviews and focus groups. Fifteen interviews were conducted with clients (n = 8 male and 7 female) who were receiving services on the units where the peers were employed, including both inpatient (n = 10) and outpatient (n = 5) units serving clients diagnosed with mood and anxiety disorders (n = 4) or schizophrenia (n = 11). Ten interviews were conducted with disciplinary staff (e.g., occupational therapists, social workers, nurses) working on the mood and anxiety unit (n = 4) and the schizophrenia unit (n = 6). Interviews with both clients and staff were semi-structured and open-ended, focused on the participants’ perceptions of the nature and consequences of their interactions with the peers, on the participants’ opinions of the value of the peer program, and on their descriptions of any challenges they saw in the program.

In addition, the research team held three focus groups: one for program developers (n = 8, all female), one for peers (n = 5, 1 male, 4 female), and one for recovery facilitators (n = 4, all female). (An additional interview was held with a program developer who was unable to attend the focus group. One staff member both participated in an interview and attended a focus group.) Program developers were invited to reflect on the origins of the program, the theory underlying its design, the impact they perceived the peers to be having, and any problems encountered. In the peer focus group participants explored their own perceptions of their work, including the value they perceived it to have, the impact they saw themselves having, and any problems they had experienced. Recovery facilitators were asked to offer their own thoughts on the peer program’s successes and challenges. All interviews and focus groups were audio-recorded and transcribed verbatim. Research team members involved in data collection wrote short memos that included both observations that would not be captured by the transcripts and their initial analytic impressions. In addition, the team met regularly during the data collection period to talk about the developing analysis and to debrief more generally about data collection activities.

The research was approved by the research ethics board at the Centre for Addiction and Mental Health; all interview and focus group participants provided written informed consent.

Analysis was concurrent with data collection and began early in the study with the development of categories for the activity tracking log. Members of the advisory committee were asked to reflect on what peers did that should be captured by the evaluation. Extensive discussion led to the identification of multiple types of work and brief definitions of these types of work, that then became the tool’s categories. When analyzing the log data gathered, the research team used the peers’ entries to calculate the amount of the peers’ time spent on each of the various types of work. The proportion of time spent on each activity was calculated based on the total time reported.

Analysis of the interview and focus group transcripts, and of the descriptive notes included in the activity logs, was conducted by the first author with multiple opportunities for consultation with the research team and the advisory committee. Using a modified grounded theory approach, the data were coded using a framework based on the work categories used in the activity log. As the analysis progressed, additional codes were developed to capture details about work processes (including types of work that had not been identified by the advisory committee), the relationships between the types of work performed and contextual conditions like peer characteristics and work settings, and the consequences of peers’ work. Visual data displays were used to organize the coded data, and also served an abductive function, suggesting further analytic questions for constant comparison. Written memos summarized key processes, conditions, and consequences. These memos were presented to the research team and the advisory committee. Their comments and questions then fed back into the developing qualitative analysis, as did questions raised by the quantitative findings from the activity logs [39]. In this way, the researchers were able to offer full descriptions of the types of work performed by peers, explore how work processes were affected by characteristics of the employment settings (including the challenges posed by these settings), and begin to theorize about the ways in which peers’ work led to certain kinds of results.

## Results

This paper focuses primarily on findings related to the first evaluation question: what are the peers doing? Because what peers do is affected by the difficulties they encounter, it also touches on some of the challenges the peers were facing. Similarly, although this paper does not focus on the question of peers’ impact, we do briefly mention findings about peers’ perceived effectiveness in order to explore how the ways they do their work may facilitate that positive impact.

### The types of work peers do

During the 11 days recorded in the logs, peers spent 56% of their time working directly with clients (direct work) and 44% doing work that supports their work with clients (indirect work). The percentages of time spent doing direct and indirect work in both inpatient and outpatient settings are shown in Tables 1 and 2.

**Table 1:** Percentage of time spent on different direct activities

	**Total**		
	**(n = 11)**		
	**mean%**	Average total number of minutes per day/person**	**SD**
Type of Activity*			
Advocacy	16.3%	33.6	37.7
Connecting to resources	36.9	78.4	67.9
Experiential sharing	68.3	153.2	82.3
Building community	33.4	80.5	110.4
Relationship building	65.3	149.3	103.4
Group facilitation	14.1	28.6	42.7
Skill building/mentoring/goal setting	38.8	79.6	70.5
Socializing/self-esteem building	63.9	142.7	104.1
Other	8.3	14.6	39.3

*Categories are not mutually exclusive.

**Number of minutes refers to total number of minutes spent on type of activity in one day, not number spent per contact.

**Table 2:** Percentage of time spent on different indirect activities

	**Total**		
	**(n = 11)**		
	**mean %**	Average total number of minutes per day/person**	**SD**
Type of Activity*			
Group planning and development	15.4%	25.0	32.9
Administration	27.2	40.0	35.8
Team communication	24.3	40.5	53.5
Supervision/training	8.3	13.6	28.8
Receiving support	10.8	24.6	37.5
Education/awareness building	10.4	23.2	50.8
Information gathering and verification	16.8	38.2	52.5
Other	11.3	20.5	31.7

*Categories are not mutually exclusive.

**Number of minutes refers to total number of minutes spent on type of activity in one day, not number spent per contact.

The specific activities that made up the general categories of work tracked in activity logs were:

#### Advocacy

Advocacy was defined in the log instructions as “addressing a problem with or barrier to accessing a service or an infringement of rights.” One participant defined advocacy more broadly as “facilitating whatever the clients want.” In practice, advocacy work encompassed both the work that peers do in fighting for what clients want and the work that they do to provide clients with the wherewithal to fight for themselves. Notes in the peers’ activity logs indicated that they perceived themselves to be engaged in advocacy work when they did things like answer questions or provide information to clients, run a peer support group, work with clients around goal setting and confidence building, and discuss stigma. Helping clients navigate the mental health system appeared to be a large component of advocacy. For example, the data contained numerous examples of peers teaching clients about the mental health system and their rights within the system. While advocacy occupied a relatively small percentage of the peers’ time on the days tracked by the logs, activities falling under the general rubric of advocacy were the focus of many interviews—particularly client interviews—suggesting that advocacy is an extremely important component of what peers do.

#### Connecting to resources

This type of work was defined in the log instructions as “connecting [client] to desired services/supports.” The data showed that peers work to connect clients to resources both inside and outside the hospital. Inside the hospital, especially on locked units, peers appeared to spend a great deal of time escorting clients off the unit, thus allowing access to valued resources like a smoke, money (at the hospital’s cash office), a cup of coffee, or a breath of fresh air. Other within hospital resources included activity groups and peer support itself. Outside the hospital, peers worked to connect clients to paid and volunteer work opportunities and other activities, to housing, to transportation, to financial resources, and to sources of peer support and other support in the community. Notes in the activity logs showed that peers consider preparing clients for connection and following up after clients have connected to be integral to this type of work.

#### Experiential sharing

Defined in the log instructions as “sharing common experiences; listening to client’s experiences and sharing one’s own experiences,” experiential sharing occupied the greatest percentage of peers’ time during the days tracked. According to the data collected through interviews and focus groups, drawing upon their own life experiences in order to share their knowledge is a common element in everything that peers do—from running support groups to speaking with clients one-on-one to educating staff. The data suggested that experiential sharing is not just a type of work but also a component of one of the key mechanisms, experience, that links what the peers do to the impact their activities have.

#### Building community

Building community was defined in the log instructions as “connecting to programs to link the client into the community…or doing things that build a sense of community for clients collectively.” In the interview and focus group data, peers’ community building activities encompassed two main aims: to establish a sense of community for clients during their involvement with a unit and to help clients make meaningful lives in the community (outside the unit or hospital). Toward the first aim, many of the peers’ activities revolved around planning and conducting groups, trips, and special events (like holiday parties) for clients. They also included outreach to and orientation of new clients to unit programming. Peers built community when they invited client participation and ran groups in ways that made people feel comfortable and welcome—for example, by providing refreshments. Toward the second aim, peers did things like check in with clients who had recently moved to new community housing and made recommendations for community-based programming. Like advocacy, building community was more salient in the interview data than it was frequent in the logs. Staff participants were particularly attuned to the first aim—ways in which peers were able to create a sense of community for clients.

#### Relationship building

This type of work was defined in the log instructions as “developing trust and rapport [with clients].” The interview and focus group data suggested that relationship building included the work of initiating relationships with clients, of establishing relationships, and of maintaining relationships. Among the specific activities that made up this type of work were the introductions of peers and clients (usually either initiated by the peer or by another staff member), conversations between peers and individual clients in which peers gave advice, acted as a sounding board, or simply listened, taking clients out to smoke or to walk, making (and keeping) appointments with clients, checking in with clients who had not been in recent contact, and visiting clients who had recently transitioned from hospital to community or vice versa. Peers also built collective relationships with groups of clients, generally in group settings. Relationship building was the second most frequent type of work accounted for in the logs, and was also very salient in the interview and focus group data.

#### Group facilitation and group planning and development

These two types of work were defined in the log instructions as “leading a group activity” and “planning or organizing a group,” respectively. Peers planned and conducted groups that were specifically focused on mental health (e.g., WRAP groups, recovery groups) and groups that might be mental health promoting, but were focused on other activities (e.g., music or art groups, exercise groups). Peers planned and facilitated groups on their own, but also worked with other staff collaboratively to develop groups and group activities and to co-facilitate groups. The work of planning and developing groups required a series of steps from conception to execution, including using models to conceive groups, designing group activities to respond to client needs and requests, finding written materials to supplement group activities, making arrangements (booking space, procuring refreshments, etc.), publicizing the group, and inviting client participation. Group work was extremely salient in the interview data, particularly among clients and particularly in inpatient settings.

#### Skill building/mentoring/goal setting

This type of work was defined in the log instructions as “developing [client] skills and goals.” The data suggested that this work related to tangible, instrumental skills and goals, such as resume writing or meditation, to issue-specific skills and goals, such as providing advice about specific clinical or government programs, and to very abstract skills and goals, like helping clients to feel hopeful. Peers worked with clients both to develop and meet clients’ personal goals—for example, pertaining to housing or employment—and to set shared goals for the peer/client relationship. Some of the specific activities that made up this type of work included the facilitation of peer support and recovery groups, one-on-one conversations in which peers provided advice and encouragement to clients—for example, coaching them about how to interact with providers or going online with them to look for volunteer work, and activities directed at building client confidence and increasing client familiarity with resources in order to facilitate informed choice.

#### Socialization/self-esteem building

Defined in the log instructions as “coaching related to social communication skills, social skills or encounters,” the data suggested that socialization and self-esteem building work was embedded in most of the direct work, both individual and group, in which peers were engaged. It was inherent in how peers initiated contact with clients and in the ways in which they worked to support and sustain their relationships with clients. In outpatient programs, this type of work often entailed making dates to meet clients in the community for coffee and conversation. In inpatient settings, it meant escorting clients off the unit for coffee or a meal or performing what clients called “good deeds” or thoughtful gestures, like buying refreshments for a group, that boost client self-esteem. This category of work was very difficult to distinguish from other types already described, especially relationship building and skill building/mentoring/goal setting.

#### Administration

The administrative work performed by peers included activities like responding to email and telephone messages, preparing for and wrapping up after groups and special events, doing odd jobs around the office (e.g., answering phones for an absent colleague), and documenting (e.g., writing progress notes). Peers also found themselves doing administrative tasks related to their employment at the hospital. Administrative work was the most frequent indirect activity tracked in the logs, but was not at all salient in the interview and focus group data.

#### Team communication

Team communication was defined in the log instructions as “team meetings or other communication with team members.” Neither the logs nor the other data suggested that peers spend much, if any, time in traditional team meetings (though there is some indication that peers in some units may be starting to attend such meetings). Most “team” communication actually took place one-on-one between peers and individual staff members. The communication activities captured by the logs included the conversations between peers and staff members related to groups or events they were working together to plan or conduct, conversations in which peers conveyed information about clients to staff, conversations in which peers went to staff to troubleshoot on a client’s behalf or to air a client’s concerns, and conversations in which peers and other staff were strategizing or debriefing about a client or group of clients. These conversations generally took place in person, though peers also used email to conduct some communications.

#### Supervision/training

This type of work was defined in the log instructions as “meetings to discuss [peer’s] performance and role or completing hospital-mandated training.” Information gathered in our focus groups suggested that peers had engaged in a number of structured training and supervision activities earlier in their employment. Perhaps because data collection took place over the summer, however, this activity had low frequency and little salience in our data.

#### Receiving support

The log instructions defined this type of work as “help seeking from ‘intentional allies’ and other colleagues.” The frequency of this type of work was very low in the logs. These data, and the interview and focus group data, suggested that peers seek only specific instrumental and task-related support—for example, the kinds of activities described in team communication—from their fellow staff members. A few peers had established good collaborative working relationships with the recovery facilitators assigned to their units. However, most peers appeared to receive only very limited personal support from colleagues, including other peers.

#### Education/awareness building

This type of work was defined in the log instructions as “education for the public and the hospital community.” The interview and focus group data presented examples of peers engaged in structured and unstructured educational activities like organizing recovery events, providing feedback on unit programming, and otherwise sharing their expertise with colleagues. Peers played a role in the hospital’s new staff orientation activities and presented on peer support in other venues. The frequency of education/awareness building work was very low in the logs. However, this was a type of work that the peers were very interested in and seems to hold great potential for expansion.

#### Information gathering and verification

Defined in the log instructions as “seeking up-to-date information about policies and public benefits to better inform clients,” this type of work was fairly frequent in the log accounts. Activities that relied on this type of work, like providing information and responding to clients’ questions about available services, supports, and opportunities, were extremely salient in the interview data, particularly in the client interviews. Peers described using the internet and attending workshops as prime sources of information. There was some suggestion in the data that peers may do a great deal of this type of work on their own time—that is, as unpaid labour.

Analysis of the interview and focus group data revealed that in addition to the work tracked in the activity logs, peers also engage in types of work that are less visible but equally important because they make possible the direct and indirect work peers do for which they are held accountable. The first of these types of work was *forging collegiality*. Just as peers strive to initiate, establish, and maintain relationships with clients, so too did they work to build collegial relationships with other staff. Toward this end, peers spent time learning the culture of the units and programs in which they were working, including learning to understand the priorities, values, knowledge, territories, constraints, and stresses of the disciplinary staff with whom they work. Peers were able to use this knowledge to make themselves useful—for example, by performing a task in order to cover for an absent or harried colleague or by intervening with a client in order to diffuse a potentially disruptive situation—and to show respect or deference to other staff—for example, by seeking advice from colleagues before implementing an idea or by passing along information about a client that a staff member needed to do his or her own job. The formal and structured collaborative work that peers did with other staff helped to promote collegiality, as did their involvement in the informal and unstructured social events (e.g., water cooler chat, holiday parties) that brought co-workers together.

Peers also performed work aimed at *legitimizing the peer role*. Indeed, the peers were acutely aware that they were “pioneers” in a new role at the hospital. Among the pressures they experienced were not only those related to doing their jobs well, but also to justifying the very existence of the job. Thus, they had to define and negotiate the peer role—what they would and would not do, what they could and could not do — in a context that mixed high expectations and stigma-based stereotyping and discrimination. Peers worked to manage expectations—their own and those of others; to avoid errors that they perceived may make the peer role vulnerable; and to “pick their battles”—judging when to respond to discriminatory words or actions (directed against clients or themselves) and when to let them go.

Both forging collegiality and legitimizing the peer role may be understood as strategies peers used to negotiate some of the challenges of the peer role. Specifically, this work becomes part of what one participant described as the “dance” peers must perform in order to respond to varying forms of individual and institutional resistance to their employment — a lack of awareness and understanding of the peer role, clashes between the peers’ philosophy and the hospital’s traditional biomedical ideology, territoriality and defensiveness on the part of some disciplinary staff, and stigma and discrimination directed toward both clients and peers.

### How peers have positive impact

The data collected to answer the evaluation’s second question, about the impact peers were having, found both clients and staff members believed that peers were having positive effects on the quality of life, and of care, for clients in the domains of information and other resources, engagement, morale, empowerment and hope. Analysis directed at exploring *how* peers were achieving these results suggested five key characteristics of the ways peers were doing their jobs that facilitated their perceived effectiveness:

#### Experience

Peers draw upon their own life experiences, especially experiences of distress, poverty, and oppression, on the one hand, and experiences of recovery and resilience, on the other. These experiences have led them to have a body of knowledge that is extremely useful to clients. The fact that they have had these experiences means that they are able to understand clients in a way that is real and empathetic. Because of their own experiences, they are able to make meaningful connections with clients.

#### Approach

Peers initiate contact with clients in a way that is respectful and calm. They encourage client engagement without pressure, and accept the nature and extent of engagement that clients wish to have. Their manner of approach appears to send a message to clients that it is about them (the clients), not about the peer or the requirements of the institution.

#### Presence

Peers are able to be with clients in a way that demonstrates genuine concern. They listen actively and pay attention to clients. Their responsiveness and kindness validates clients. On restricted units the physical presence of peers allows clients greater freedom and more access to valued resources than they would otherwise enjoy.

#### Role modeling

Peers serve as symbols and examples to both clients and other staff. They provide “someone to look up to” for clients who are seeking ways of living that will help them to meet their goals. For staff, they stand as exemplars of “recovery in action,” and their skills and knowledge serve as examples to staff looking for a new way in which to work. Role modeling is something that happens naturally and informally in private interactions, but there are also times when it is used formally in public forums.

#### Collaboration

In almost of all of their activities, peers are working with other people, either clients or staff. While peers stand somewhat outside the institutional hierarchy, and thus are able to be “independent,” they also lack any real power, so much of what they can accomplish requires the cooperation of others. Their role is very much defined by their connectedness. Thus, peers spend a lot of time promoting or supporting that cooperation—doing things like information sharing, finding allies, etc.

There are two other process characteristics that are most apparent in the “invisible” work that peers do: *challenge* and *compromise*. Challenge refers to the promise (or threat) of change that peers bring to the status quo. Challenge is embedded in the very existence of the peer role, but is especially apparent in the work of advocacy and education/awareness building that they do. Compromise represents the ways in which peers must restrain or moderate challenge in order to maintain their legitimacy.

## Discussion

Experience, approach, presence, role modeling, collaboration, challenge, and compromise can be seen as the tangible enactments of peers’ philosophy of work. That is, the ways in which peers “do” support reflect their commitment to a strengths-based, respectful, and non-judgmental approach that is grounded both in their own life experiences and in a nuanced understanding of the broader systems in which these experiences are embedded.

## Limitations

The results of this evaluation should be considered in the light of its limitations. One of its major limitations is the extent of its generalizability in either its qualitative or quantitative components. The experiences of the participants for this evaluation may not necessarily be representative of those of other similar programs. The clients and staff who participated in the evaluation may not be reflective of all clients and staff.

In addition, the activity log data were collected for a short period of time. As a result, the results are subject to greater influences by anomalies. For example, because this study was conducted during the summer months, clinical staff and clients may have taken vacations. This would change the typical activities. Future research should look at collecting activity logs for longer periods of time to minimize the effects of seasonal fluctuations and other temporary variations.

Nevertheless, the types of work identified in this study, as well as our findings about the challenges and the impact of peer support, are consistent with those found in other research. This consistency provides some confidence that despite the limitations of size and setting inherent in a formative evaluation conducted in one facility over a short period of time, the results may have some generalizability. In particular, the findings from this study should be useful in providing some of the descriptive detail other investigators have identified as necessary to improving the specification of the peer role.

As we have reviewed, the literature suggests that problems can arise when peers work in positions that lack clear roles, well defined job tasks, clear expectations, and accurate job descriptions; when they are placed in settings that provide inadequate preparation for the teams that they will be working with; and when there are insufficient structures or policies in place to support the contribution of peers to the system, guide recruitment and selection, and define the peer position [4,33-35]. Better job descriptions would be beneficial not only to peers, but to teams and organizations.

The information gathered from this evaluation will contribute to a better understanding of the peer support worker position, the skills required and the types of expectations that could define successful fulfillment of the role.

### A Job description

The findings of this evaluation lead us to propose a peer job description:

#### Tasks/duties

This is a non-clinical role. The peer will work collaboratively with clients, co-workers and the community. He/she will advocate on behalf of clients and help clients to navigate the health and social services systems. The candidate will work closely with clients to address problems and answer questions, gather and provide information and advice, and connect clients to resources and to the community. The peer will meet clients both in the hospital and in the community. He/she will initiate, establish and maintain relationships with clients while developing trust and rapport. The peer will act as a coach and mentor, and help clients to set goals and work toward developing skills. He/she will share and discuss common experiences with clients. The peer will help to build a collective sense of community for clients, and help clients to create meaningful lives in the community. The peer will be responsible for planning, organizing, developing, leading and facilitating group activities, including education and awareness building efforts. The peer will complete administrative duties, such as sending and receiving email and telephone calls, and completing required documentation. He/she will also complete any training required for the position. The candidate will communicate and work collaboratively with team members, attend team meetings, and meet with supervisors to discuss performance.

#### Qualifications

Candidates must have experience of mental health and/or addiction problems. They should also have knowledge/familiarity of the mental health and social service systems, and an understanding of client rights. The incumbent should possess a holistic perspective of “health.” Candidates will demonstrate the ability to work effectively in a wide range of settings with people from diverse backgrounds, including clients and co-workers. Candidates should be comfortable working either one-on-one or in group settings, must possess excellent interpersonal skills and should be able to adapt to changing situations. Active involvement in the community and a willingness to collaborate with others is required. Candidates should possess excellent communication skills. They should be comfortable with public speaking and facilitation of group workshops or activities. They should also be comfortable coaching others, and possess negotiation skills. Candidates should be well-organized and have some experience planning and designing events and activities. Computer skills would be an asset.

The job description suggests that candidates for the peer position require more than experience with mental health and/or addiction problems and familiarity with the mental health and social service systems through which clients must navigate. Expectations of the job indicate that strong communication skills are of paramount importance to the position. Because of the collaborative nature of the position, peers must be able to work in changing situations with a diverse group of people, interacting either in groups or individually. They should be actively involved in the community and willing to take on leadership and public speaking activities. Coaching and negotiation skills are assets for this position, as are planning, organization and computer skills. These job requirements describe the high level of skills necessary to be an effective peer support worker. They also highlight the areas in which there are opportunities for training for peer support workers before they enter their positions as well as ongoing training to help them hone their skills.

It is also important to note that although job descriptions are extremely useful, they should not be considered fixed. Programs must be prepared to assess and reassess their own needs and local context, including the skills and experience of the peer workforce. Peer programming will evolve, as will the individuals employed as peers, and job descriptions should be adjusted to reflect this evolution. The job description in this article may not be appropriate for all settings, but it will contribute to a better understanding of the peer support worker position, the skills required, and the types of expectations that could define successful fulfillment of the role.

## Conclusions

Appropriate job descriptions are essential to the success of the job incumbent because they help to ensure that the recruitment and selection process is executed effectively and that the best candidate is selected for the job. They also guide the goals and activities of the incumbent once he or she is hired. The findings of this evaluation led us to propose a general peer job description that may be useful to organizations seeking to develop peer support programming. A successful peer will have qualifications beyond having had experience with mental health and/or addiction problems. A relevant job description should specify the other types of skills and experiences that characterize a well-qualified and effective candidate. In this way, it can help facilitate the integration of peers into their multi-disciplinary work teams and add legitimacy to the work of peers.

## Competing interests

The authors declare that they have no competing interests.

## Authors’ contributions

NJ led the conception, design, data acquisition, analysis and interpretation of the qualitative data. LT collaborated on the data acquisition and the qualitative analysis. CSD collaborated on the design, acquisition, analysis and interpretation of the qualitative data and led the analysis of the quantitative data. All authors contributed to writing and all read and approved the final manuscript.

## Pre-publication history

The pre-publication history for this paper can be accessed here:

http://www.biomedcentral.com/1472-6963/12/205/prepub
